# Milk-Gelling Properties of Proteases Extracted from the Fruits of *Solanum Elaeagnifolium* Cavanilles

**DOI:** 10.1155/2022/4625959

**Published:** 2022-10-18

**Authors:** Carolina Nájera-Domínguez, Néstor Gutiérrez-Méndez, Diego E. Carballo-Carballo, María Rosario Peralta-Pérez, Blanca Sánchez-Ramírez, Guadalupe Virginia Nevarez-Moorillón, Armando Quintero-Ramos, Antonio García-Triana, Efren Delgado

**Affiliations:** ^1^The Graduate School, Graduate Program in Chemistry, Chemistry School, Autonomous University of Chihuahua, Mexico; ^2^Consumer and Environmental Sciences, College of Agricultural, New Mexico State University, New Mexico, USA

## Abstract

There is little information on the milk coagulation process by plant proteases combined with chymosins. This work is aimed at studying the capability of protease enclosed in the ripe fruits of *Solanum elaeagnifolium* (commonly named trompillo) to form milk gels by itself and in combination with chymosin. For this purpose, proteases were partially purified from trompillo fruits. These proteases had a molecular weight of ~60 kDa, and results suggest cucumisin-like serine proteases, though further studies are needed to confirm this observation. Unlike chymosins, trompillo proteases had high proteolytic activity (PA = 50.23 U_Tyr_ mg protein^−1^) and low milk-clotting activity (MCA = 3658.86 SU mL^−1^). Consequently, the ratio of MCA/PA was lower in trompillo proteases (6.83) than in chymosins (187 to 223). Our result also showed that milk gels formed with trompillo proteases were softer (7.03 mPa s) and had a higher release of whey (31.08%) than the milk gels clotted with chymosin (~10 mPa s and ~4% of syneresis). However, the combination of trompillo proteases with chymosin sped up the gelling process (21 min), improved the firmness of milk gels (12 mPa s), and decreased the whey release from milk curds (3.41%). Therefore, trompillo proteases could be combined with chymosin to improve the cheese yield and change certain cheese features.

## 1. Introduction

Historically, the cheese was made using the rich-chymosin liquid extracted from the abomasal tissues of young ruminants. These abomasal extracts contain proteases like chymosin, which are highly efficient in milk clotting. For instance, calf rennet contains up to 95% of chymosin (EC 3.4.23.4) and a minor fraction of pepsin (EC 3.4.23.1), though this proportion depends on the animal's age [1]. However, calf rennet is currently used only to manufacture specific dairy products. Instead, microbial [2] and recombinant chymosin proteases are used most to manufacture cheese nowadays. Speaking of recombinant chymosins, these are commonly expressed in bacteria (*E. coli*), yeast (*Kluveryomyces lactis*), or molds (*Aspergillus niger* var. *awamori*). The recombinant chymosins are becoming very popular among cheesemakers since their high milk-clotting properties, vegetarian approval, kosher certification, and protection of animal rights. Nevertheless, the downside of recombinant chymosins is that some countries and consumers have concerns or negative perceptions of genetically modified organisms (GMOs) [1, 3, 4]. Therefore, many researchers have turned their eyes to plant proteases to replace chymosin-based rennets [5].

All plants contain proteases, and plant genomes encode hundreds of different proteases [6]. However, most plant proteases hydrolyze milk proteins excessively, generating weak milk gels or not forming gels [7]. Only a few plant proteases can induce suitable milk gelation [7]. For example, proteases from date [8], papaya [9], ginger [10], grape [11], sunflower seed [12], orange blossom [13], ficus [14], fennel [15], and *Cynara cardunculus* flowers [16] have shown caseinolytic and milk-clotting activity. As far as our knowledge is concerned, only the cardosin protease extracted from *Cynara cardunculus* flowers is used for industrial-scale cheese production [7]. One main reason for the limited usage of plant proteases is their excessive caseinolytic activity and poor milk-clotting capacity [4, 12, 17]. Another reason is that most plant proteases have not been completely characterized or studied. Accordingly, they could be misused in cheese production (i.e., wrong temperature, pH, or amount of protease), getting unsatisfactory results [7].

*Solanum elaeagnifolium* (trompillo) is an endemic plant from the southwest United States and northeast Mexico. Trompillo fruits have been used for decades to manufacture Asadero cheese [18], an artisanal pasta filata-type cheese that shares similarities with mozarella cheese [19]. The small trompillo fruits (1-2 cm diameter) appear during the summer and fall. A single trompillo plant produces up to 60 fruits. These fruits are green and fleshly in the summer, but they dehydrate in the winter, turning yellowish, brown, or black. The dried fruits release their many seeds (60 to 120 seeds per fruit) effortlessly [18, 20, 21], as shown in Table 1.

Until now, trompillo proteases have not been thoroughly characterized. However, previous studies have revealed that the green fleshy and overripe black fruits from trompillo plants do not present proteolytic activity (PA) or milk-clotting activity (MCA). Only the dried and yellowish fruits showed MCA, and this activity was slightly higher in the seeds (3125 SU mL^−1^) than in the fruit peels (2564 SU mL^−1^). The proteases in the yellowish fruits have not been thoroughly identified. Still, in a previous study, we found that trompillo proteases might have a molecular weight between 50 to 70 kDa, and they could be serine proteases (at least one) [22]. Similarly, Aquino-Favela et al. [23] identified trompillo proteases as cucumisin-like serine proteases with a molecular weight of 66 kDa. In an early study, Nasr et al. described proteases from trompillo fruits as thermostable [24] since these plant proteases were active up to 84°C. Despite the scientific knowledge on trompillo proteases, little is known about their milk-gelling properties and the similarities or differences with chymosin, the typical protease used in cheese making. Therefore, this work is aimed at studying the capability of proteases enclosed in the ripe fruits of *Solanum elaeagnifolium* to form milk gels by themselves and in combination with chymosin.

## 2. Materials and Methods

### 2.1. Vegetable Material

**Figure 1:**
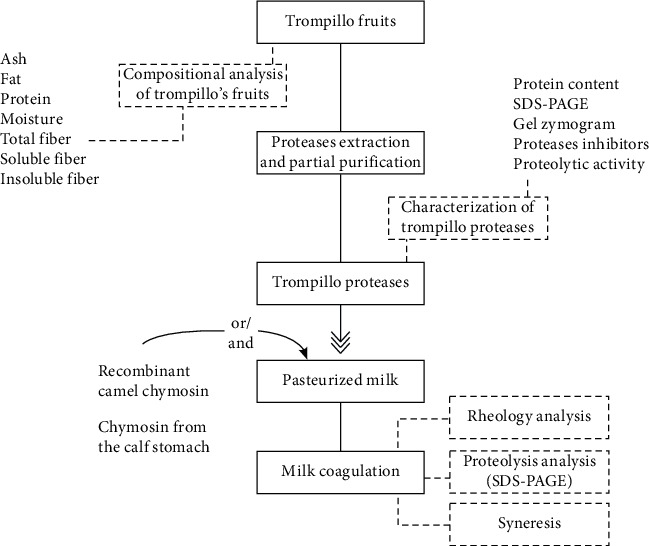
Schematic diagram of the methodology used in this study.

Fruits from *Solanum elaeagnifolium* Cavanilles plants (trompillo) were collected in Chihuahua, Mexico, during the fall and winter of 2019. First, the yellow and dry fruits (Table 1) were grounded with a knife mill (Thomas Wiley Fisher, model 3383-L19, Swedesboro, NJ, USA) and sieved through a 0.841 mm mesh (mesh number 20). Next, the gross composition of the fruit powder was determined according to the AOAC methods: ash 942.05, fat 948.22, protein 960.52, moisture 934.01, total, soluble, and insoluble fiber 991.43 (Figure 1). Finally, the fruit powder was stored in hermetic-sealed plastic bags at -20°C.

### 2.2. Proteases

This study used two types of chymosins and the protease extracted and partially purified from trompillo fruits (details below). One of the chymosins used was recombinant camel chymosin expressed in *Aspergillus niger* var. *awamori* with EC 3.4.23.4 (Chy-Max M1000, Chr. Hansen, Horsholm, Denmark). The other chymosin was bovine chymosin from the calf's stomach with EC 3.4.23.4 (Sigma Aldrich St. Louis, MO).

### 2.3. Extraction and Partial Purification of Proteases from Trompillo's Fruits

The fruit powder was mixed in a ratio of 1 : 3 (*w*/*v*) with sodium acetate buffer (0.05 M, pH 5) and added with NaCl (50 g L^−1^). This mixture was kept under magnetic stirring for 24 hours at 4°C. Then, the liquid was filtered through three layers of gauze and centrifuged at 3000 × g for 20 min at 4°C. The resulting supernatant was filtered (Whatman No. 1 filter paper) and stored at -20°C until use. Such supernatant was considered the crude extract (CE) and was later used in the partial purification of proteases.

The CE was subject to protein precipitation using ammonium sulfate at various saturation levels. The saturation processes were conducted under continuous stirring (600 rpm) at 0°C for 30 minutes. Proteins precipitated were recovered by centrifugation (3200 × g, 20 min at 4°C) and resuspended (1 : 3 *v*/*v*) in sodium acetate buffer (0.05 M, pH 5) [24, 25].

### 2.4. Characterization of Trompillo Proteases Partially Purified

First, protein content in the fraction with trompillo proteases partially purified was quantified according to Bradford's method [26]. Then, this fraction was analyzed by sodium dodecyl sulfate-polyacrylamide gel electrophoresis (SDS-PAGE) as described elsewhere [22], and a gel zymogram was performed to confirm the presence of proteases in this fraction. The gel used for this purpose was an SDS-polyacrylamide gel added with casein at 20 g L^−1^. This gel was run at 100 volts (Mini-PROTEAN II cell, Bio-Rad, CA) until the bromophenol blue dye marked reached the gel bottom. Next, the gel was washed with Triton X-100 (25 g L-^1^) for 30 minutes and submerged into a tris-buffer solution (50 mM tris, pH 7, 5 mM CaCl_2,_ and 0.2 M NaCl) for 48 h at 37°C. Lastly, the gel was stained with Coomassie blue dye and slightly washed with the tris-buffer solution. The clear zone upon gel staining indicates proteolytic activity [27].

The proteolytic activity of trompillo proteases was measured by Lowry's method [28] with slight modifications [29]. Briefly, 25 *μ*L from the purified protease fraction was added to 130 *μ*L of casein solution (6 g L^−1^) and incubated at 30°C for ten minutes. The reaction was stopped with 130 *μ*L of 100 mM trichloroacetic acid (TCA). The mixture was centrifuged at 3000 × g for 15 min at 10°C to separate the hydrolyzed (supernatant) and nonhydrolyzed (sediment) caseins. Then, a supernatant aliquot of 250 *μ*L was mixed with 625 *μ*L of Na_2_CO_3_ (0.5 M) and 125 *μ*L of Folin-Ciocalteu reagent (0.2 N). This mixture was incubated for 30 min at 37°C and read at 760 nM (Biotek, Elx808, Winooski, VT, USA). Sample blank was prepared similarly but substituting the protease solution with distilled water. The calibration curve was built using tyrosine standards (5.5, 11, 27.5, and 55 *μ*M). The reaction velocity (*v*) was calculated with Equation (1), where [Tyr_protease_] and [Tyr_blank_] are the calculated concentration of tyrosine in the protease solution and the blank. Meanwhile, *t* represents the time of reaction (10 min). The proteolytic activity (PA) was calculated with Equation (2), where *V*_enzyme_ is the volume of enzyme used (25 *μ*L), and *V*_reaction_ is the volume of reaction (155 *μ*L). One unit of enzyme activity (U_Tyr_) was the amount of enzyme that releases one micromole of tyrosine equivalents per minute of reaction.

(1) $$\begin{array}{c} V=\frac{\left[{\text{Tyr}}_{\text{protease}}\right]-\left[{\text{Tyr}}_{\text{blank}}\right]}{t}, \end{array}$$

(2) $$\begin{array}{c} PA=\frac{v\times V_{\text{reaction}}}{V_{\text{enzyme}}}. \end{array}$$

In addition, the proteolytic activity of trompillo proteases was evaluated under the presence of different protease inhibitors to identify possible types of proteases. The inhibitors used were 10 mM ethylene diamine tetra-acetic acid (EDTA) as a metalloprotease inhibitor, 10 mM phenylmethylsulfonyl fluoride (PMSF) as a serine-protease inhibitor, 0.01 mM pepstatin A as an aspartic-protease inhibitor, and 0.01 mM trans-epoxysuccinyl-L-leucylamido (4-guanidino)-butane (E-64) as a cysteine-protease inhibitor.

### 2.5. Milk-Gelling Properties of Trompillo Proteases, Chymosins, and their Combination

The milk-gelling properties of proteases were studied following changes in milk viscosity during the coagulation process. The tests were conducted in a rotational rheometer (Anton Paar, Rheolab QC, Austria) with a concentric cylinder geometry and a conical tip (CC39, ø 3.899 cm, 132°). Before the assays, pasteurized milk (% fat 3.07 ± 0.06, % protein 2.68 ± 0.026, and % lactose 3.82 ± 0.040) was warmed at 30°C, added with CaCl_2_ (0.2 g L^−1^), and poured into the rheometer cup (70 mL). Then, each protease was added to the milk at different volumes to ensure all milk-coagulation experiments would happen within a 40-minute time frame. The volume of each protease added into the milk was as follows: recombinant camel chymosin = 15 *μ*L (1.30 mg protein mL^−1^), chymosin from calf stomach = 20 *μ*L (0.63 mg protein mL^−1^), and trompillo proteases = 27 *μ*L (0.63 mg protein mL^−1^). Additionally, one treatment included the mixture of 15 *μ*L of recombinant camel chymosin with 27 *μ*L trompillo proteases, obtaining this mix with a protein concentration of 0.86 mg protein mL^−1^. All the rheological tests were performed under a constant shear rate of 50^-s^ at 30°C. Three replicates from each treatment were analyzed rheologically using the methodology previously described.

**Figure 2:**
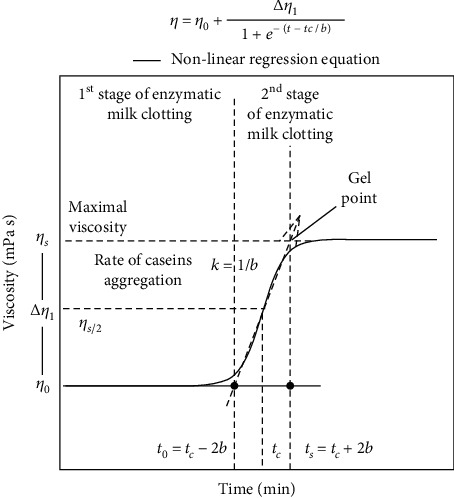
Sigmoidal four-parameters equation used to analyze the rheological behavior of milk clotted with bovine chymosin, recombinant camel chymosin, or trompillo proteases. *η*_*s*_=viscosity at gel point, *η*_0_=initial viscosity, *η*_*s*/2_=half of the maximum viscosity, *t*_*c*_=time to reach half maximum viscosity, *k*=rate of caseins aggregation, *t*_0_=time to start the caseins aggregation, and *t*_*s*_=time to form the milk gel.

Data were collected for 80 minutes with the rheometer software (Rheoplus V3.20, Ostfildern, Germany) and fitted to the four-parameter sigmoidal function (Figure 2). In this equation (Equation (3)), *η*_0_ is the initial viscosity or milk viscosity, Δ*η*_1_ is the increase of viscosity during gel formation, *t* is the time of incubation, *tc* is the time to reach half maximum viscosity, and *b* is the reciprocal value for the rate of casein aggregation (*k*).

(3) $$\begin{array}{c} \eta =\eta_{0}+\frac{∆\eta_{1}}{1+e^{-\left(t-t_{c}/b\right)}}. \end{array}$$

The milk-clotting activity (MCA) for each protease solution was calculated with Equation (4), where *V*_milk_ and *V*_protease_ were the volumes of milk and protease used, respectively. The time to reach the gel point or maximal viscosity (*t*_*s*_ = *t*_*c*_ + 2*b*) was calculated from Equation (3). Finally, the MCA values were expressed in soxhlet units (SU) per milliliter of protease solution, where one SU is the volume of milk (mL) that can be clotted by one milliliter of protease solution in 40 min at 30°C.

(4) $$\begin{array}{c} \text{MCA}=\frac{V_{\text{milk}}}{V_{\text{protease}}}\times \frac{1\text{mL protease}}{40\;\min}\times \left(t_{c}+2b\right). \end{array}$$

### 2.6. Milk-Protein Hydrolysis during Gel Formation

During the gelling process, the breakdown of milk proteins by trompillo proteases was monitored at time intervals. For this purpose, sterile tubes were filled with 70 mL of pasteurized milk added with CaCl_2_ (0.2 g L^−1^). Next, 27 *μ*L (0.63 mg protein mL^−1^) of the plant-derived protease were incorporated into the milk and incubated at 30°C. At ten-minute intervals, one milliliter of milk or milk gel was taken from each tube and mixed with one milliliter of TCA to stop protease activity. These milk samples were analyzed by SDS-PAGE and proteolytic activity described in Section 2.4.

### 2.7. Syneresis in Milk Gels

By triplicate, 35 mL of milk with CaCl_2_ (0.2 g L^−1^) were poured into sterile tubes and added with the corresponding protease solution (see details above). The tubes were incubated at 30°C for 80 minutes for milk gel formation. Then, the tubes were centrifuged for ten minutes at 10°C and 300 × g. The volume of whey released after centrifugation was measured and weighted. Syneresis was expressed as the relative percentage of whey released from the milk gels after 80 minutes of incubation [30]. Protein content in the whey released by the milk gels was analyzed by Bradford's method [26]. In addition, the whey released by milk gels was subject to SDS-PAGE analysis to investigate the milk proteins and peptides in the whey.

### 2.8. Statistical Analysis

A randomized one-factor design was used to compare the plant-derived protease and the chymosins. The experimental factor (*τ*_i_) was the type of protease with the following levels: (1) plant-derived protease, (2) recombinant camel chymosin, and (3) bovine chymosin. Each treatment was replicated three times (*n* = 3). The data collected were analyzed by one-way analysis of variance (ANOVA) and Tukey-Kramer multiple range test (*α* = 0.05).

## 3. Results and Discussion

### 3.1. Extraction and Partial Purification of Trompillo Proteases

The powder obtained from the yellow fruits of *Solanum elaeagnifolium* contained mostly carbohydrates, primarily fiber. Ten percent of trompillo's composition was protein (Table 1). Gutiérrez-Méndez et al. have reported protein content ranging from 4.5 to 13.8% [18]. Composition discrepancies arise chiefly because of differences in the water content of fruits. The fruits of *Solanum elaeagnifolium* are produced during the summer (one single plant produces up to 60 fruits). At first, the fruits are green with white patches and fleshly with significant water content [21]. These greenish fruits have no proteolytic or milk-clotting activity, suggesting that trompillo's protease is in a zymogen form in this maturity stage [22]. Trompillo fruits become dehydrated and turn yellow-orange during the hot summer (Table 1). Such ripened and dry fruits are brittle, break easily, release many flat seeds (up to 120 seeds per fruit) [31], and have a high proteolytic and milk-clotting activity [22]. A previous study revealed that the proteolytic activity in dehydrated trompillo fruits is either in fruit peels or seeds [22]. Therefore, we milled whole dehydrated yellow-orange fruits to recover and purify trompillo's protease.

**Figure 3:**
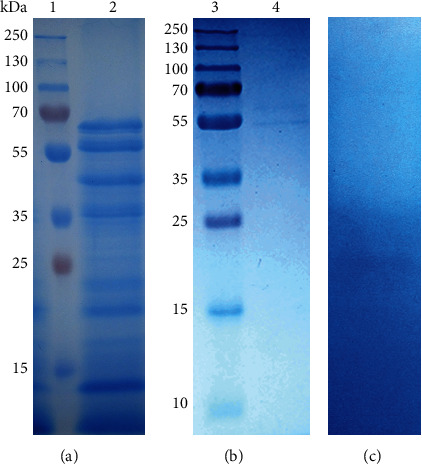
SDS-PAGE analysis of the crude extract from *Solanum elaeagnifolium* fruits (a), and partially purified proteases from the crude extract (b), with its proteolytic zymogram (c). Lane 1=molecular weight marker, lane 2=crude extract, lane 3=molecular weight marker, lane 4=fraction recover sulfate ammonium precipitation (50%) and gel filtration on Sephadex G-100.

The partial purification of trompillo proteases started by obtaining a CE from the dried fruits of Trompillo. Eleven proteins were observed in this CE (Figure 3(a)) with the following molecular weights: 66, 57, 45, 36, 34, 26, 22, 19, 15, 11, and 8 kDa. After CE protein precipitation with ammonium sulfate at diverse saturation levels, it was detected that the precipitate obtained at 50% saturation had the highest proteolytic activity. This fraction was further separated by size-exclusion chromatography (SEC) using Sephadex G-100 (Sigma Aldrich St. Louis, MO) and sodium acetate buffer (0.05 M, pH 5) as mobile phase. The selected fraction from SEC with the maximum proteolytic activity showed two proteins with 57 and 66 kDa of molecular weight (Figure 3(b)), agreeing with previous results identifying trompillo proteases (50 to 70 kDa) [22]. In addition, the proteolytic zymogram analysis confirmed the presence of proteases in this protein fraction by forming a clear zone in the polyacrylamide-casein gel (Figure 3(c)). According to these results, trompillo proteases share similarities with milk-clotting proteases from other *Solanum* plants, such as those obtained from *Solanum dubium* fruits. Like trompillo proteases, *S. dubium* proteases (66 kDa) have high milk-clotting activity and are used in the manufacture of artisanal cheese (in Sudan) [32, 33]. Besides *Solanum dubium*, another nine *Solanum* species (endemic to Cameroon) have been investigated for milk-clotting proteases: *S. acuelustrum*, *S. aethiopicum*, *S. anomalum*, *S. cerasiferum*, *S. dasyphyllum*, *S. indicum*, *S. nigrum*, *S. nodiflorum*, and *S. terminale* [34]. Unfortunately, the milk-clotting proteases from these plants have not been purified and characterized.

The assays with protease inhibitors revealed the possible presence of a serin protease on the partially purified fraction of trompillo proteases. The aspartic-protease inhibitor (pepstatin A) and the cysteine-protease inhibitor (E-64) reduced the proteolytic activity of trompillo proteases by 55 and 66%. In contrast, the PMSF and EDTA inhibitors decreased the proteolytic activity of trompillo proteases by 79 and 70%, respectively. The EDTA is considered a metalloproteinase inhibitor, and the PMSF is a serine-protease inhibitor. Unlike the metalloproteinases (EC 3.4.24) [35], the plant serine proteases (EC 3.4.21) are stable and active in alkaline conditions (pH 7 to 11) and at high temperatures (30 to 80°C) [36, 37], like previously reported for trompillo proteases (47 to 81.5°C) [38]. In addition, other authors have identified trompillo protease [18, 39] as a cucumisin-like serine protease (~60 kDa). The cucumisin-like serine proteases exhibit intense caseinolytic activity, have a molecular mass of ~60 kDa, and some show high milk-clotting activity. For instance, the cucumisin-like serine protease obtained from *Solanum dubium* (66 kDa) [32], *Cucumis melo* (66-68 kDa) [40], and *Ficus religiosa* (80 kDa) [41]. Considering all these results, we concluded that the partially purified fraction of Trompillo proteases presented at least one cucumisin-like serine protease.

### 3.2. Proteolytic and Milk-Clotting Activity of Chymosins and Trompillo Proteases

**Figure 4:**
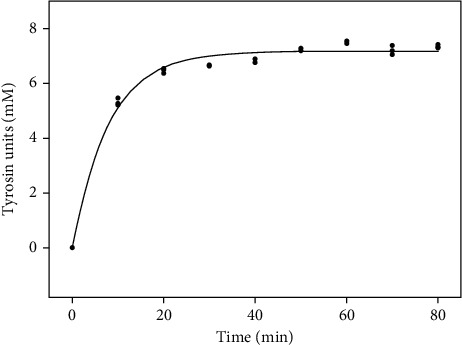
Kinetic of caseins hydrolysis by trompillo proteases at 30°C and pH 7. Values represent the average of triplicates (*n* = 3).

The specific proteolytic activity of trompillin (U_Tyr_ mg^−1^ protein) was almost twice as high as the recombinant camel chymosin and the chymosin from the calf stomach (Table 2). The kinetic of caseins hydrolysis by trompillo proteases (at 30°C and pH 7) is showed in Figure 4. Other plant-derived proteases like ficin (from fig tree latex), dubiumin (from *Solanum dubium* fruits), and papain (from papaya fruit) have also shown superior proteolytic activity than chymosins [30, 33]. Overall, it has been well documented that most plant-derived proteases have an intense caseinolytic activity, contrasting with the low proteolytic activity of chymosins [4, 7].

trompillo proteases had greater milk-clotting activity (MCA) than other plant-derived proteases such as ficin, bromelain [30], and dubiumin [32]. However, the proteases from trompillo fruits had a much lower capacity to clot the milk than the recombinant and calf chymosins (Table 2). As far as we know, no plant-derived protease has superior MCA than chymosin, not even the cardosins A and B from *Cynara cardunculus* [42], which are plant proteases used in the industrial manufacture of cheese [7]. Indeed, most plant proteases cannot coagulate the milk, chiefly because they hydrolyze caseins extensively, avoiding gel networks. Few plant proteases can induce milk gelation, though these vegetable coagulants usually present a very low ratio of milk-clotting activity to proteolytic activity (MCA/PA) [7, 12, 43]. The MCA/PA ratio for trompillo proteases was more prominent than reported for other plant proteases like ficin and bromelain [30, 44] but lower than observed in the recombinant camel chymosin or the chymosin from the calf stomach (Table 2). These large MCA/PA ratios indicate that chymosins produce a very low (and specific) proteolysis on milk proteins, which induces the coagulation of a large volume of milk [45]. Therefore, chymosin remains the preferred coagulant in cheese manufacturing.

### 3.3. Formation of Milk Gels by Using Chymosins and Trompillo Proteases

Trompillin proteases generated softer milk gels than the two chymosins (Table 3). In addition, the maximal viscosity (*η*_s_) reached in the milk clotted with trompillo proteases was lower than *η*_*s*_ observed in the milk coagulated with the recombinant chymosin and the calf chymosin. Interestingly, when the recombinant chymosin was mixed with trompillo proteases, the milk gels attained a maximal viscosity (*η*_*s*_) superior to the milk gels coagulated only with recombinant chymosin (Table 3).

**Figure 5:**
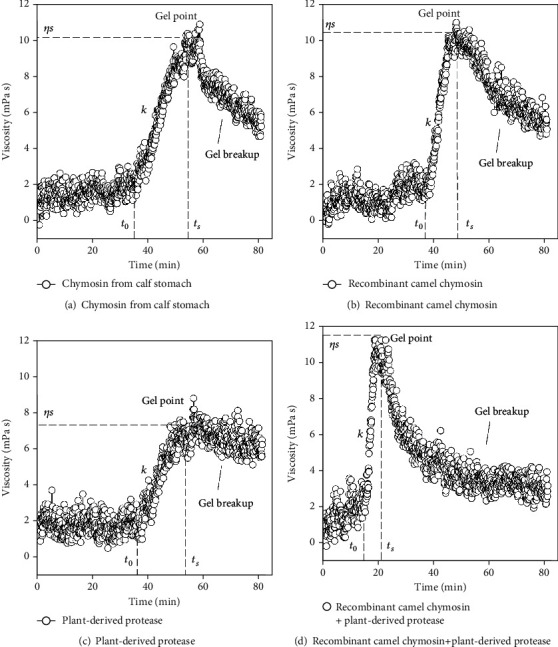
Rheological analysis of milk gelation induced by different proteases at 30°C. *η*_*s*_=viscosity at gel point, *k*=rate of caseins aggregation, *t*_0_=time to start the caseins aggregation, and *t*_*s*_=time to form the milk gel.

The enzyme volume for each protease was adjusted to trigger milk protein aggregation in approximately 40 minutes (*t*_0_~40 min). Trompillo proteases and the two chymosins had a *t*_0_ of around 38 minutes, with no difference between them (Figures 5(a)–5(c), and Table 3). However, the aggregation of milk proteins happened at different rates (*k*), depending on the protease used. For example, the *k*-value for trompillo proteases was about half the value of the two chymosins (Table 3). Consequently, the time to induce milk gelation once milk proteins started their aggregation (Δ*t* = *t*_*s*_ − *t*_0_) was longer in the milk clotted with the plant proteases than in milk clotted with chymosins. Overall, the gelling process in milk added with the trompillo proteases was slower than observed for the recombinant chymosin and the calf chymosin. Nevertheless, the plant proteases combined with chymosin accelerated the milk-gelling process significantly.

**Figure 6:**
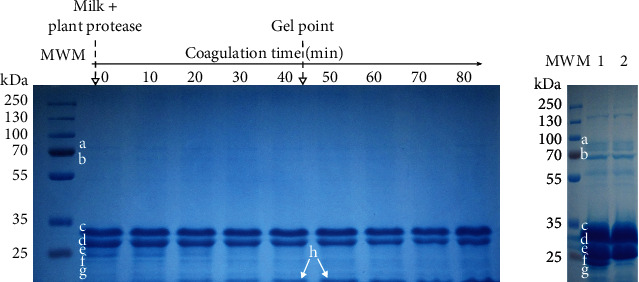
Hydrolysis of milk proteins during gel formation at 30°C under the action of different proteases. (a) Milk clotted with trompillo proteases. (b) Milk clotted with recombinant camel chymosin (lane 1) and chymosin from calf stomach (lane 2); samples were taken after 80 minutes of incubation. MWM=molecular weight marker, a=transferrin, b=serum albumin, c=*β*-caseins, d=*α*_s1_-caseins, e=*κ*-caseins, f=*γ*-caseins, g=*β*-lactoglobulin, h=hydrophobic peptides~14 kDa.

According to the SDS-PAGE analysis, the plant proteases hydrolyzed more milk proteins during milk coagulation than chymosins (Figure 6). Unlike chymosins (Figure 6(b)), trompillo proteases hydrolyzed all caseins, particularly the *κ*-caseins, which were almost totally depleted within the first 30 minutes (Figure 6(a)). However, the plant proteases did not hydrolyze whey proteins like *β*-lactoglobulins, perhaps because of their native globular conformation (Figure 6(a)). It is also worth mentioning that some peptides derived from caseins hydrolysis by trompillo proteases were held in the milk gel matrix. These peptides had a molecular weight of ~14 kDa and were probably poorly water-soluble since they did not migrate to the whey during gel syneresis.

Milk coagulation by chymosin has been extensively studied, and there is a consensus that chymosin-induced coagulation occurs in two stages [4, 46]. In the first stage, chymosins hydrolyze the *κ*-casein fragments protruding from casein micelle surfaces. The *κ*-caseins expose 69 (out of 169) amino acids on the micelle surface, providing stabilization through steric and electrostatic forces. The hydrolysis of *κ*-caseins protruding from casein micelles decreases in half the negative micelle charge (-20 mV) and minimizes the steric repulsion between casein micelles. Consequently, millions of casein micelles (10^14^-10^16^ micelles per mL of milk) aggregate in the presence of Ca^2+^ and form gel networks (stage two) [45–48]. It has been estimated that only 60% of *κ*-caseins must be hydrolyzed to induce casein micelles aggregation, which explains the effectiveness of chymosins gelling milk [49]. Unlike plant proteases, chymosins have low proteolytic activity and an elevated capability to coagulate ruminant milk. These chymosin features have physiological and nutritional significance for newborn ruminants. The low proteolytic activity avoids immunoglobulins hydrolysis, and milk coagulation stimulates an appropriate function for developing stomachs [4]. This work confirmed that chymosins produce limited and specific hydrolysis on milk proteins (Table 2, Figure 6(b)). Such low proteolytic activity induces the formation of very cohesive milk gels in a short time (Table 3, Figures 5(a) and 5(b)).

Trompillo proteases were less effective in promoting casein micelles aggregation than chymosins. Plant proteases depleted almost all *κ*-caseins in 40 minutes (Figure 6(a)), although most *β*-caseins and *α*_s1_-caseins remained unhydrolyzed after 80 minutes (Figure 6(b)). Furthermore, the rate of casein aggregation (*k*) and the final gel viscosity (*η*_s_) was lower in the milk gels clotted with Trompillo proteases than in those coagulated with chymosins (Table 3). It is worth mentioning that these results are not exclusive to Trompillo proteases. It has been reported that other plant proteases like ficin and bromelain induce slow milk coagulation and the formation of soft milk gels [30]. These results suggest that plant proteases completely deplete the negative charges on casein micelle surfaces by hydrolyzing *κ*-caseins exhaustively. The total elimination of repulsive forces induces the aggregation of casein micelles but avoids the formation of salt bridges between Ca^2+^ and the negative residues from *κ*-caseins. Consequently, casein micelles aggregate slowly (low *k*) and weakly (low *η*_s_), similar to what happens in acid-induced milk coagulation, where micelles aggregate by screening the negative charges instead of by salt bridges [50, 51]. In contrast, chymosins hydrolyze *κ*-caseins partially (~60%) and create multiple salt bridges between Ca^2+^ and negative sites on para-*κ*-caseins [48], forming thus a strong and cohesive gel network (Figures 5(a) and 5(b), and Table 3).

Interestingly, when the recombinant camel chymosin was mixed with the plant protease, the casein micelles aggregated faster (high *k*) and stronger (high *η*_*s*_) than when these enzymes were used alone (Figure 5(d), Table 3). This effect was probably related to simultaneous specific and nonspecific hydrolysis of *κ*-caseins. In this sense, chymosins hydrolyze *κ*-caseins at a particular peptide bond between Phe_105_ and Met_106_, releasing a macropeptide and leaving a para-*κ*-casein fragment attached to the casein micelle (Figure 6(b)). Such specific hydrolysis decreases the repulsive and steric impediments, favoring casein micelles aggregation. However, the random or nonspecific hydrolysis carried out by trompillo proteases (Figure 6(a)) helped minimize the repulsive forces and the steric impediments between casein micelles quickly and efficiently. As a result, strong gels (high *η*_s_) were formed briefly (Figure 5(d), Table 3).

### 3.4. Syneresis of Milk Gels Clotted with Trompillin and Chymosins

**Figure 7:**
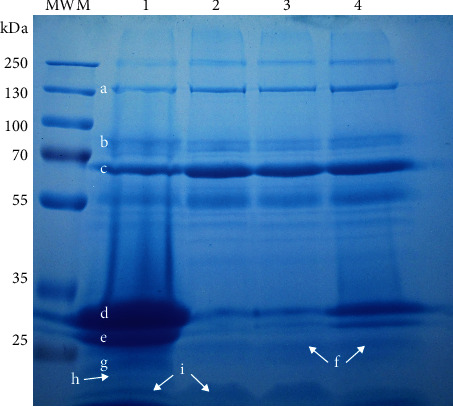
Proteins observed in the whey released by milk gels clotted with Trompillo proteases (lane 1), recombinant camel chymosin (lane 2), bovine chymosin (lane 3), and the mixture (1 : 1 ratio) of recombinant camel chymosin with Trompillo proteases (lane 4). MWM=molecular weight marker, a=immunoglobulins, b=lactoferrin, c=serum albumin, d=*β*-caseins, e=*α*s_1_-caseins, f=*κ*-caseins, g=*γ*-caseins, h=hydrophilic peptides~18 kDa, i=*β*-lactoglobulin.

The percentage of whey released after 80 minutes was substantially higher for the milk gels clotted with trompillo proteases than for chymosin-induced gels. In addition, the whey released from milk gels clotted with plant proteases had nine times more protein than the whey from chymosin-induced gels (Table 4). The proteins in the whey released from trompillo-induced gels were mostly caseins or partially hydrolyzed caseins, whey proteins, and peptides of ~18 kDa (Figure 7). Mixing the plant proteases with the recombinant camel chymosin minimized this excessive syneresis and migration of caseins. As a result, the milk gels clotted with such proteases mixture had a low percentage of syneresis and the whey released had a little protein (Table 4).

Milk gels coagulated with other plant proteases like ficin and bromelain have also shown higher syneresis than chymosin-clotted milk gels [30]. Unlike chymosins, plant proteases hydrolyze the *κ*-caseins protruding from the casein micelles but also the *β*-, *α*_s1_-, and *α*_s2_-caseins at nonspecific cleavage sites (Figures 6(a) and 7). Such extensive and random hydrolysis induces the formation of weak gel networks with low *η*_*s*_ value (Figure 5(c)), unable to hold much water or casein fragments in their structure (Table 4). However, the extensive and random hydrolysis generated by plant proteases combined with chymosin's low and specific activity led to strong milk gel structures with high *η*_*s*_ value (Figure 5(d)). These chymosin-trompillo-induced milk gels hold a more considerable amount of water (Table 4), probably because of their ordered casein micelles arrangement. Furthermore, trompillo-derived peptides likely helped increase the water retention inside the milk gel structure [52].

## 4. Conclusions

The ripen fruits of *Solanum elaeagnifolium* showed proteases of ~60 kDa, and results suggest cucumisin-like serine proteases, though further studies are needed to confirm this observation. These plant proteases had lower milk-clotting activity to proteolytic activity ratio (MCA/PA) than chymosins. In addition, the milk gels formed with trompillo proteases were softer and had a higher release of whey than chymosin-induced gels. Overall, our results coincide with those reported for other plant proteases and lead us to assume that most plant proteases, including trompillo proteases, cannot substitute the commercial chymosin-based milk coagulants. However, combining chymosin with the plant protease sped up the gelling process, improved the firmness of milk gels, and decreased the whey release from milk curds. Therefore, trompillo proteases could be combined with chymosins to enhance the cheese yield and change certain features like cheese texture and probably cheese melting.

## Figures and Tables

**Table 1:** Compositional analysis of the powder obtained from yellow and dry fruits of *Solanum elaeagnifolium* plants.

Component	Mean^1^ ± SD^2^	Other authors^4^	
	g 100 g^−1^		
Moisture	6.3 ± 0.06	10.5	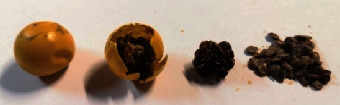
Lipids	6.2 ± 0.26	3.4	
Ashes	3.7 ± 0.01	5.6	
Proteins	10.1 ± 1.41	12.0	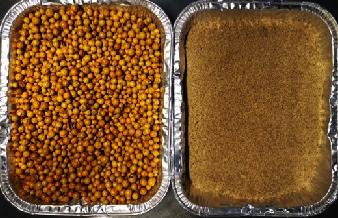
Carbohydrates^3^	73.7 ± 1.53	68.2	
Total fiber	34.1 ± 1.42	48.4	
Insoluble fiber	26.3 ± 1.52	53.8	
Soluble fiber	7.8 ± 0.10	2.9	

^1^Average of three replicates. ^2^SD=standard deviation. ^3^Estimated by difference. ^4^Average composition reported by diverse authors [18].

**Table 2:** Milk-clotting and proteolytic activities of proteases used in this study.

Parameter	Recombinant camel chymosin	Chymosin from the calf stomach	Trompillo proteases^1^
MCA, (SU mL^−1^)	6120.2 ± 223.3^a^	4397.4 ± 33.9^b^	3658.8 ± 251.1^c^
Sp-MCA, (SU mg) protein^−1^	4697.2 ± 154.3^b^	7064.7 ± 194.1^a^	841.0 ± 55.2^c^
PA, (UTyr mL^−1^)	32.6 ± 1.5^b^	19.6 ± 0.5^b^	535.6 ± 10.6^a^
Sp-PA, (UTyr mg) protein^−1^	24.8 ± 1.0^c^	30.5 ± 0.8^b^	50.2 ± 1.0^a^
Ratio MCA/PA	187.7 ± 15.7^b^	223.7 ± 8.4^a^	6.8 ± 0.5^c^

^1^Plant-derived protease from the fruits of *Solanum elaeagnifolium*. MCA=milk-clotting activity expressed in soxhlet units per milliliter (SU mL^−1^). Sp-MCA=specific milk-clotting activity expressed in soxhlet units per milligram of enzyme (SU mg^−1^). PA=proteolytic activity expressed in tyrosine units per milliliter (U_Tyr_ mL^−1^). Sp-PA=specific proteolytic activity expressed in tyrosin units per milligram of enzyme (U_Tyr_ mg^−1^). ^a,b,c^Mean values in the same row with different superscripts differ (ANOVA, Tukey-Kramer test, *P* < 0.05). Data represent the average of three replicates (*n* = 3).

**Table 3:** Rheological behavior of milk clotted with different proteases at 30°C.

Parameter	Recombinant camel chymosin	Chymosin from the calf stomach	Trompillo proteases^1^	Recombinant chymosin+trompillo proteases^1^
*η*_0_, (mPa s)	1.2 ± 0.3^a^	1.1 ± 0.3^a^	1.7 ± 0.1^a^	1.8 ± 0.2^a^
*η*_*s*_, (mPa s)	10.1 ± 0.6^b^	9.1 ± 0.8^b^	7.0 ± 0.2^c^	11.9 ± 0.4^a^
Δ*η*, (mPa s)	8.9 ± 0.5^ab^	7.9 ± 0.5^b^	5.2 ± 0.1^c^	10.1 ± 0.6^a^
*k*, (mPa s) min^−1^	1.1 ± 0.2^ab^	1.0 ± 0.7^ab^	0.4 ± 0.1^ab^	1.7 ± 0.3^a^
*t*_0_, min	38.9 ± 1.8^a^	37.6 ± 0.3^a^	37.3 ± 2.8^a^	16.4 ± 1.4^b^
*t*_*s*_, min	48.3 ± 2.7^a^	46.2 ± 0.3^a^	51.9 ± 3.5^a^	21.9 ± 2.2^b^
Δ*t*, min	9.4 ± 0.9^b^	8.6 ± 0.5^b^	14.6 ± 1.3^a^	5.5 ± 1.0^c^

^1^Plant-derived protease from the fruits of *Solanum elaeagnifolium*. *η*_0_=initial viscosity, *η*_*s*_=maximal viscosity, Δ*η*=increase of viscosity during gel formation (*η*_*s*_ − *η*_0_), *κ*=rate of caseins aggregation (1/b), *t*_0_=time to start caseins aggregation (*t*_*c*_ − 2b), *t*_*s*_=time to reach the maximal viscosity (*t*_*c*_ + 2b), Δ*t*=time to induce milk gelation once caseins started their aggregation (*t*_*s*_ − *t*_0_). ^a,b,c,d^Mean values in the same row with different superscripts differ (ANOVA, Tukey-Kramer test, *P* < 0.05). Data represent the average of three replicates (*n* = 3).

**Table 4:** Whey separation in milk gels clotted at 30°C with different proteases.

Parameter	Recombinant camel chymosin	Chymosin from the calf stomach	Trompillo proteases^1^	Recombinant chymosin+trompillo proteases^1^
Milk^2^, (g)	36.2 ± 0.3^a^	37.4 ± 0.7^a^	36.0 ± 0.5^a^	36.3 ± 1.0^a^
Whey^3^, (g)	1.6 ± 0.2^a^	1.7 ± 0.1^a^	11.2 ± 0.7^a^	1.2 ± 0.1^a^
Milk gel, (g)	34.5 ± 0.3^a^	35.7 ± 0.6^a^	24.8 ± 0.2^b^	35.1 ± 1.1^a^
Syneresis, %	4.5 ± 0.5^b^	4.4 ± 0.2^b^	31.0 ± 1.7^a^	3.4 ± 0.4^b^
[P], (g 100 mL^−1^)	0.137 ± 0.002^b^	0.138 ± 0.002^b^	0.938 ± 0.042^a^	0.164 ± 0.015^b^

^1^Plant-derived protease from the fruits of *Solanum elaeagnifolium*. ^2^Milk added with the protease solution and CaCl_2_ at 0.02% *w*/*v*. ^3^Whey released after 80 minutes of incubation at 30°C and subsequently centrifuged for 10 minutes at 10°C and 300 × *g*. [P]=protein content in the whey. ^a,b,c,d^Mean values in the same row with different superscripts differ (ANOVA, Tukey-Kramer test, *P* < 0.05). Data represent the average of three replicates (*n* = 3).

## Data Availability

Readers can access the data supporting the conclusions of this study on the following link: https://osf.io/km3x5/?view_only=fca9d4a641204dd18b5ecede7abe5d5d.
